# Effect of Geometrical Parameters on the Mechanical Performance of Bamboo-Inspired Gradient Hollow-Strut Octet Lattice Structure Fabricated by Additive Manufacturing

**DOI:** 10.3390/mi15050583

**Published:** 2024-04-28

**Authors:** Junxian Ge, Yu Song, Zhenyu Chen, Yuhao Zhuo, Tongzheng Wei, Chen Ge, Yuang Cheng, Ming Liu, Qingbo Jia

**Affiliations:** 1 School of Mechanical and Electrical Engineering, Soochow University, Suzhou 215131, China; 2 State Key Laboratory of Electromechanical Integrated Manufacturing of High-Performance Electronic Equipments, Xidian University, Xi’an 710071, China

**Keywords:** additive manufacturing, laser powder bed fusion, gradient hollow-strut lattice structure, compression strength, energy absorption

## Abstract

Hollow-strut metal lattice structures are currently attracting extensive attention due to their excellent mechanical performance. Inspired by the node structure of bamboo, this study aimed to investigate the mechanical performance of the gradient hollow-strut octet lattice structure fabricated by laser powder bed fusion (LPBF). The effect of geometrical parameters on the yield strength, Young’s modulus and energy absorption of the designed octet unit cells were studied and optimized by FEA analysis. The hollow-strut geometrical parameters that deliver the best mechanical property combinations were identified, and the corresponding unit cells were then redesigned into the 3 × 3 × 3 type lattice structures for experimental evaluations. Compression tests confirmed that the designed gradient hollow-strut octet lattice structures demonstrated superior mechanical properties and deformation stability than their solid-strut lattice structure counterparts. The underlying deformation mechanism analysis revealed that the remarkably enhanced bending strength of the gradient hollow-strut lattice structure made significant contributions to its mechanical performance improvement. This study is envisaged to shed light on future hollow-strut metal lattice structure design for lightweight applications, with the final aim of enhancing the component’s mechanical properties and/or lowering its density as compared with the solid-strut lattice structures.

## 1. Introduction

Natural cellular materials, such as wood, cork and bone, have been used for centuries, and their structures were mimicked and applied in modern structural materials, including honeycomb and foam structures [1]. The lattice structure is one form of cellular material that differs from (a) trusses or frames due to its millimeter or micrometer scales and (b) foams due to the regular repeating of the unit cell structures [2,3]. As a typical example, strut-based lattice structures have been widely used in various fields, including aerospace, automobile, biomedical implants and acoustic parts, due to their excellent mechanical strength and energy absorption capabilities [4,5,6].

Generally, the mechanical response of lattice structures can be divided into two types, i.e., the stretching-dominated and the bending-dominated [7]. Under the applied force, the structure dominated by stretching bears the tension and compression of the struts, while the structure dominated by bending experiences the bending of the struts. Therefore, the structures dominated by stretching usually show a high stiffness-to-weight ratio, while structures dominated by bending have good energy absorption characteristics [8,9]. The octet structure belongs to the former, which endows the octet-truss lattice structure with exceptionally high specific energy absorption, constant plateau stress (the stress between the initial yield and densification stage) as well as zero plastic Poisson ratio [10]. On such basis, the possibility of enhancing the octet topology by adjusting the nodal fillets and geometries of edges, corners and faces on external boundary layers has been extensively explored by previous researchers [11,12,13].

As a unique subset of lattice materials, the hollow-strut lattice materials provide a new design space to further tailor the mechanical and/or functional properties of the lattice structures [14,15]. For example, Zhao et al. developed a hollow prismatic strut for the BCC lattice structure, which achieved significantly enhanced elastic modulus (~598–1460% improvement) and changed its deformation modes from bending-dominated to stretching-dominated by tailoring the inner hollow parameters [16]. Moreover, the hollow-strut lattice structure design had little influence on its shear deformation behaviors. Noronha et al. studied the hollow-strut FCC lattice structure with a relative density of about 8–16%, and it exhibited yield strength and elastic modulus at the upper empirical limits of its solid-strut counterparts [17]. Though the density of the hollow-strut lattice materials is significantly lower than the solid-strut counterparts with similar mechanical performance, the reduced wall thickness and the high geometrical complexity of the hollow-strut octet structure become a serious issue for the traditional manufacturing processes.

With the widespread application of additive manufacturing (AM), the fabrication of complex structures with high mechanical performances is now becoming realistic. As one of the commonly studied AM techniques, laser powder bed fusion (LPBF) utilizes high laser energy to melt and consolidate metal powder beds according to a certain cross-section of the part CAD model in a layer-by-layer manner [18,19,20]. To date, LPBF has been regarded as the most preferable technology for metal lattice structure manufacturing, which is normally not easy to achieve by traditional manufacturing methods [21]. Specifically, one of the fascinating developments is that the aforementioned lightweight lattice structures can be fabricated in high-fidelity by LPBF with the unit cell length scale down to micrometers [22,23]. Moreover, the successful application of low-density aluminum and titanium alloys to the LPBF process also expanded the exploration space for lightweight and high mechanical performance lattice structure manufacturing [24,25,26].

As one of the most studied biological organisms, bamboo with hollow and node structure characteristics have stimulated numerous advanced engineering structure design and application [27,28]. Herein, the hollow structure of bamboo normally enhances the inertia moment while the bamboo node resists lateral shear and improves stress dissipation, which eventually elevates the axial and lateral strength as well as the energy absorption capabilities of the thin-walled tubes during compressive loading. Inspired by the natural characteristics of bamboo, this study proposed a gradient hollow-strut octet lattice structure design to achieve improved strength-to-weight ratio and energy absorption capability. The effect of geometrical parameters of the gradient hollow-strut design on the unit cell mechanical strength was firstly optimized by numerical simulations. The compressive stress–strain relationships and energy absorption behaviors of the optimized lattice structures were then evaluated by experimental studies, while the inherent deformation modes and failure characteristics were systematically discussed. The current study is envisaged to provide a new pathway towards high performance lightweight structure design and application.

## 2. Structure Design, Materials and Methods

### 2.1. The Structure Design Strategy

**Figure 1:**
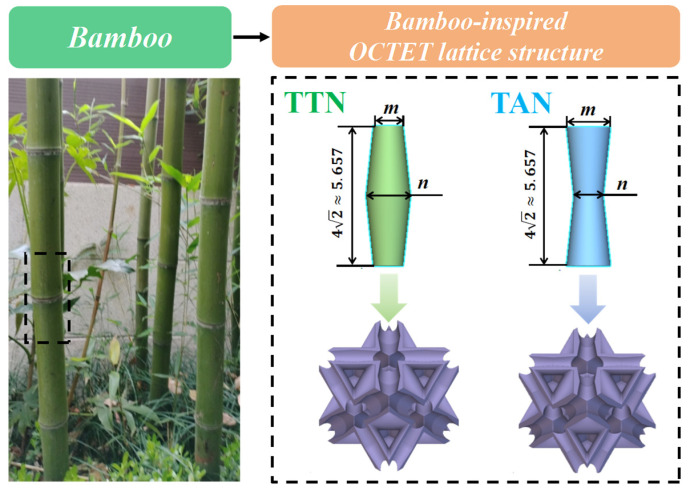
Schematic illustration of the gradient hollow-strut octet structure design strategy.

As schematically illustrated in Figure 1, the typical bamboo structure mainly consists of a hollow pillar connected by nodes at a certain distance. Herein, the node structure provides strong mechanical support to resist high bending stress induced by wind or snow. Inspired by the bamboo node, we designed two types of gradient solid struts to mimic the outer and the inner cross-section shapes of the node structure. Herein, the radii of the top and middle of the gradient solid strut were set as *m* and *n*, respectively. The top radius (*m*) is smaller for the TTN (tapered away from the node) strut and larger for the TAN (tapered towards the node) strut as compared with the middle radius (*n*). The gradient hollow-strut octet unit cells (named TAN and TTN) were obtained by subtracting the uniform cross-section of the solid-strut octet unit cells (named OCTET) from their TAN and TTN equivalents. It is worth noting the designed unit cell size was kept constant at 8 × 8 × 8 mm^3^, while the respective relative density was all set as 20%. This was determined by considering the lightweight design principles, the LPBF processability as well as the sample size requirements for experimental evaluations.

The LPBF processability of the gradient hollow struts has greater complexity compared to the solid struts, as the former requires the external topology to be uniform and structurally robust while the internal profiles must also be uniform and large enough to avoid powder occlusion. Moreover, the effective hollow-strut wall thickness of no less than 0.3 mm must also be guaranteed due to the LPBF manufacturing limits. To simultaneously satisfy such requirements, we set the top and middle radius of the gradient hollow struts as shown in Table 1 and Table 2 for TTN and TAN unit cells, respectively. The listed architectures have different shape parameter (*k*) values ranging from 0.1 to 0.3, and *k* is defined as the absolute value difference between *m* and *n*. Furthermore, we purposely added a 0.32 mm radius arc at the strut node area to further enhance the deformation stability and mechanical properties of the designed structure. The optimized unit cells were then redesigned into the 3 × 3 × 3 type lattice structures for experimental studies.

### 2.2. Finite Element Analysis

The finite element analysis (FEA) was utilized to analyze the compressive stress–strain behaviors and the resultant energy absorption characteristics of the developed unit cells and lattice structures using the ABAQUS software (version 2021). During the compression process simulation, the samples were placed between two rigid plates, and the load was applied by pressing the plates toward the sample center plane. The contact region between the plate and the sample was set as a hard contact property. Herein, the material elastic modulus and yield strength were determined by experimental tensile tests. A mesh convergence study was carried out to select the appropriate mesh size for modeling and a triangle mesh element with a size of 0.1 mm was chosen. Such mesh size can capture the geometric details of the designed gradient hollow-strut structures and provide accurate solutions without prolonging the calculation time.

### 2.3. Sample Preparation and Microstructural Observation

The fabrication of the studied lattice structure samples was conducted on a commercial EP-M150 LPBF machine (E-Plus-3D, China), using the gas-atomized AlSi10Mg alloy powders as the raw materials. The optimized laser process parameters, including laser power of 370 W, laser scanning speed of 1300 mm/s, hatch distance of 110 μm and layer thickness of 30 μm, were applied to fabricate the bulk tensile samples as well as the developed lattice structures. The above parameters can guarantee fabricated samples with a porosity level below 0.5%.

The surface morphology of the prepared lattice structure was observed using a VHX-7000 3D optical microscope (OM) (Keyence, Japan). Before observation, the lattice structure samples were cleaned by an ultrasonic vibrator in water several times until no powder was observed. The microstructure of the printed bulk sample was characterized by an advanced Symmetry S (Oxford, UK), electron backscattered diffraction (EBSD) detector installed in the Gemini 300 (Zeiss, Germany), scanning electron microscope (SEM), and the sample was etched using a solution containing 30 mL alcohol,16 mL hydrochloric acid and 5 mL nitric acid.

### 2.4. Mechanical Property Evaluation

An AG-IS 50 kN (Shimadzu, Japan) universal mechanical testing machine was used for uniaxial tensile and compression tests to obtain the mechanical properties of the bulk samples and the designed lattice structures, respectively. Two repeated tests of each sample condition were conducted to verify the data repeatability. The tensile and compression tests were performed under a constant cross-head moving velocity of 0.5 mm/min and 2 mm/min, respectively, and the corresponding load-displacement data were recorded to calculate the stress–strain curves. For the lattice structure compression tests, the deformation process was recorded by a digital camera analyzing its deformation modes and failure behaviors.

## 3. Results and Discussion

### 3.1. AlSi10Mg Sample Microstructure and Tensile Properties

**Figure 2:**
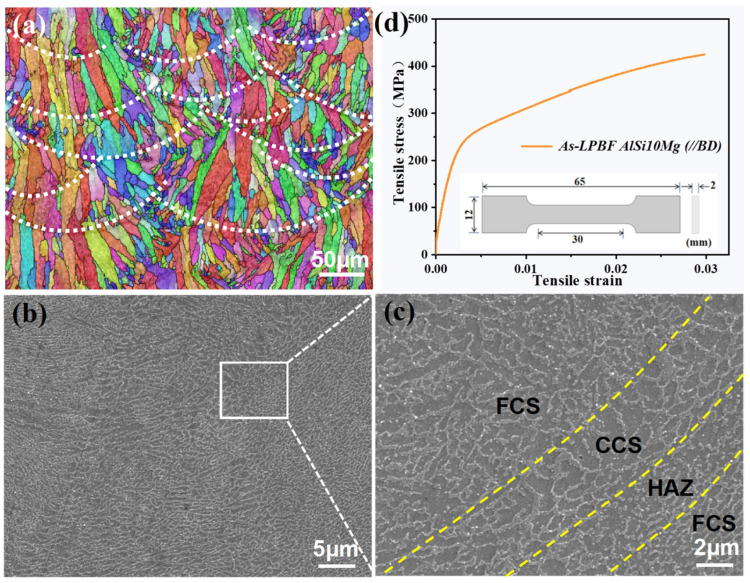
Microstructure and tensile properties of the LPBFed AlSi10Mg alloy sample. (**a**) The columnar grain structures revealed by EBSD, (**b**) fine cellular structures, (**c**) different microstructure regions within a molten pool and (**d**) engineering stress–strain curves of the LPBFed AlSi10Mg sample. The inserted picture in (**d**) illustrates the geometrical parameters of the tensile sample.

Figure 2 presents the microstructure and tensile properties of the LPBFed AlSi10Mg alloy sample. During the LPBF process, the sample is built by stacking the molten pools in a track-by-track and layer-by-layer manner, in which the ultrafast cooling rate normally triggers the formation of unique microstructures. The alloy mainly consists of columnar grain structures due to the epitaxial grain growth, and this is induced by the directional heat dissipation towards the molten pool boundary areas [29]. Thanks to the ultrafast cooling rate, fine cellular structures were observed within the columnar grains in Figure 2b, differing the microstructures of LPBFed AlSi10Mg samples from their conventional casting counterparts (typically with long eutectic Si needles) [30]. Higher magnification image revealed three distinct microstructure zones within a single molten pool, including the molten pool core areas with fine cellular structures (FCSs), the melt pool boundary areas with relatively coarse cellular structures (CCSs) and the heat-affected zone (HAZ) areas with fractured cellular structures (Figure 2c). Such microstructure variance within the individual molten pools can be attributed to the local solidification condition and/or the intrinsic heat treatment effect induced by the layer-by-layer building process. The readers are suggested to refer to [29] for more details. Figure 2d shows the tensile stress–strain curves of the LPBFed AlSi10Mg alloy sample. The LPBFed AlSi10Mg alloy sample demonstrated outstanding tensile properties with a measured yield strength of 258.88 ± 3.48 MPa, surpassing its casting counterparts with typical yield strength of only about 100 MPa. Moreover, the elongation to fracture and the Young’s modulus of the fabricated sample were determined as 2.98 ± 0.1 % and 73.63 ± 2.43 GPa, respectively. Obviously, the extraordinarily fine microstructure within the melt pool contributed to its high mechanical strength, and the above properties were utilized for the following numerical studies.

### 3.2. Structural Parameter Optimization of the Designed Unit Cells

**Figure 3:**
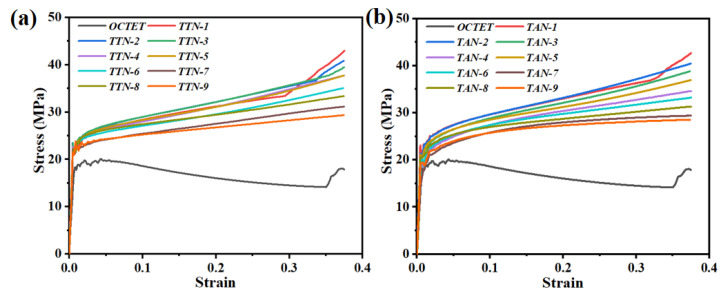
The FEA-simulated stress–strain curves of the developed (**a**) TTN and (**b**) TAN gradient hollow-strut unit cells and the solid-strut counterparts.

To achieve the most balanced mechanical property combinations between strength, Young’s modulus and energy absorption, the designed unit cells were subjected to FEA simulations. The simulated stress–strain curves as a function of the geometric parameter (*m* and *n*) values of the gradient hollow strut are presented in Figure 3. Obviously, all the studied TTN and TAN structures exhibited higher strength values than their solid-strut counterparts under the same relative density. Moreover, it can also be found that the geometric parameter variance in the hollow-strut topology design played an important role in the mechanical performance of the developed TTN and TAN unit cells. The stress basically decreased with the decrement of the hollow-strut radius for both types of unit cells.

**Figure 4:**
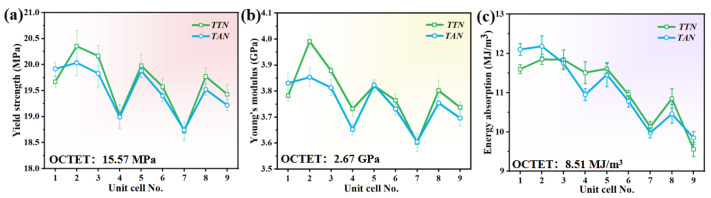
The derived compressive mechanical performance from Figure 3 of (**a**) yield strength, (**b**) Young’s modulus and (**c**) energy absorption of the developed TTN and TAN unit cells.

To gain intuitive understanding of the property variance between the developed unit cells, the mechanical performance, including elastic modulus, yield stress and the energy absorption, was derived from the above stress–strain curves and displayed in Figure 4. Herein, it is worth noting that the yield strength is chosen as the stress at a compression strain of 0.2%, while the energy absorption (*EA*) that represents the ability of a material to withstand load without catastrophic failure can be expressed as [31]

(1) $$EA(\varepsilon )=\int_{0}^{\varepsilon_{1}}\sigma d\varepsilon$$

where *ε* is the compression strain, *σ* is the compression stress, and *ε*_1_ is the onset strain of densification. As shown in Figure 4, the mechanical performance of the developed unit cells exhibited up-and-down trends with the change in the hollow-strut geometrical parameters. Specifically, the maximum values were harvested when the shape parameter *k* equals 0.1 for all the studied octet unit cells. On the other hand, the TTN hollow-strut structure demonstrated relatively higher strength values than the TAN hollow-strut structure, except those at both larger *m* and *n* values. Nevertheless, we can easily find that the TTN-2 and TAN-2 structures showed the highest yield strength, Young’s modulus and energy absorption among all the studied unit cells.

### 3.3. Experimental Evaluations of the LPBFed Lattice Structures

**Figure 5:**
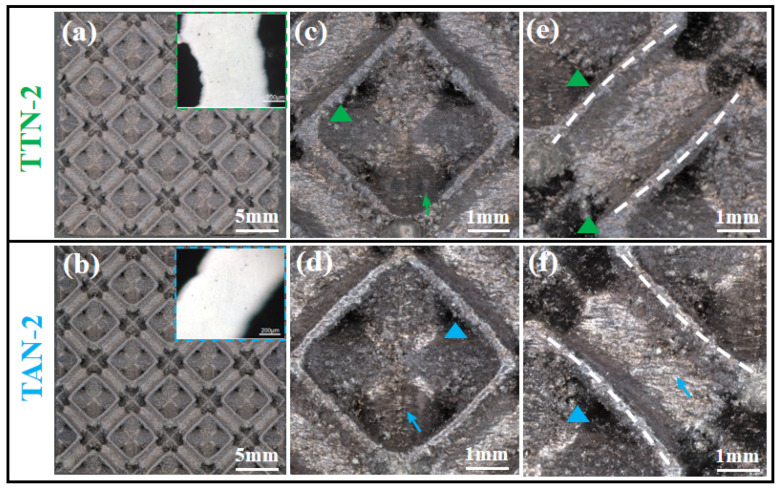
Surface characteristics of the fabricated (**a**,**c**,**e**) TTN-2 and (**b**,**d**,**f**) TAN-2 lattice structure samples. (**a**,**b**) The overall morphology and the respective porosity level of the strut, (**c**,**d**) the magnified view of the node area and (**e**,**f**) the magnified view of the hollow-strut area.

The above optimized TTN-2 and TAN-2 unit cells were then redesigned into 3 × 3 × 3 type lattice structures and subjected to LPBF manufacturing. The overall and local surface morphology features of the fabricated lattice structures are shown in Figure 5. The LPBFed TTN-2 and TAN-2 lattice structures basically demonstrated sound consistency and integrity as compared with the CAD model (Figure 5a,b). The inserted pictures in Figure 5a,b confirmed the low porosity level (typically below 0.5%) of the solid material of the strut structure. Moreover, the node area showed good formability, while the gradient size change of the hollow struts was also captured, confirming the high manufacturing fidelity of the LPBF process (Figure 5c,d). However, as indicated by the triangle symbols, careful observation revealed that the partially melted metal powders were bonded to the down-surface areas in both lattice structures. For the unsupported overhang structure, part of the strut down-surface is built on the loose powders, which is easy to cause the unstable molten pool and the adhesion of the partially melted powder to the strut down-surface areas. In addition, as indicated by the arrows, the step-like features were also observed on both the inner and outer surfaces of the hollow strut. The above surface defects can cause mechanical property inconsistency to the LPBFed lattice structures, and future surface quality improvement needs to be conducted by either tailoring the process parameters or applying post-surface treatment processes.

**Figure 6:**
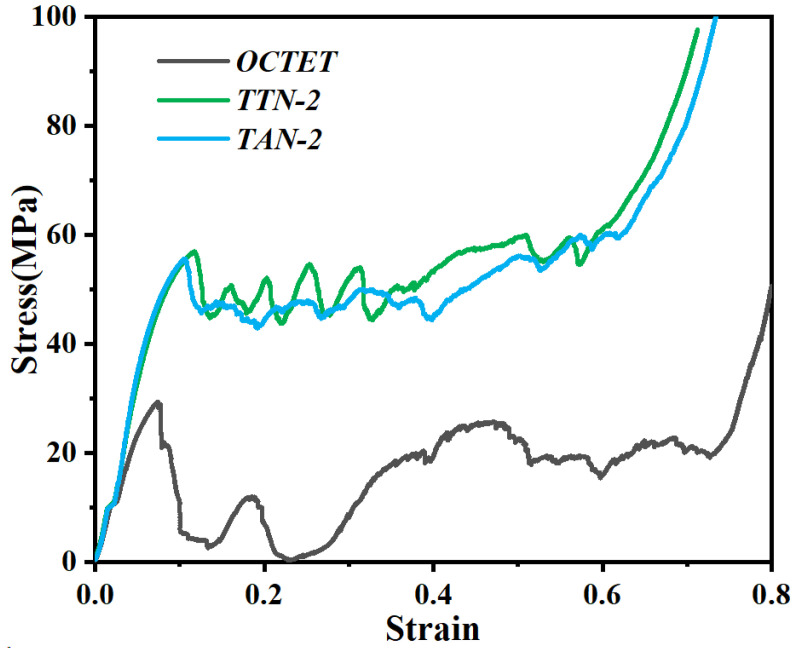
The experimental compressive stress–strain curves of the studied lattice structures.

Figure 6 shows the experimentally determined compressive stress–strain curves of the LPBF fabricated octet lattice structures. Herein, the mechanical performance of the gradient hollow-strut octet lattice structures was compared with their solid-strut counterparts under the same relative density. It is worth noting that the slight curve deviation during the initial compression testing (at a stress level of about 10 MPa) may be caused by the uneven surface of the lattice structures. All the studied samples exhibited three deformation stages, including the linear elastic stage I, the plateau stage II (characterized by multiple damage collapse mechanisms) and the densification stage III (where the stress–strain curve rises sharply) [32]. As can be seen in Figure 6, the optimized gradient hollow-strut lattice structures exhibited remarkably higher yield stress than the solid-strut sample. The compression stress of the solid-strut lattice structure dropped rapidly after the initial yielding, indicating the local severe fracture or collapse of the lattice structure, while it subsequently experienced significant fluctuations until the onset of the densification strain. On the contrary, the gradient hollow-strut TTN-2 and TAN-2 lattice structures demonstrated relatively stable deformation behavior with only slight stress oscillation during the plateau deformation stage. Furthermore, the TTN-2 lattice structure exhibited relatively higher yield stress and plateau stress than the TAN-2 structure.

**Figure 7:**
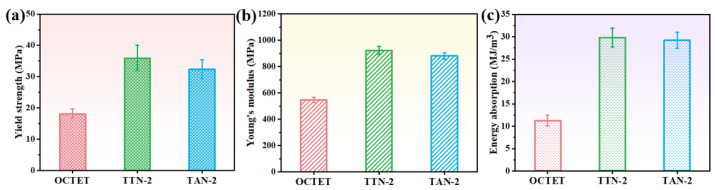
The calculated (**a**) yield strength, (**b**) Young’s modulus and (**c**) energy absorption of the LPBFed lattice structures.

Figure 7 displays the calculated mechanical properties of the LPBFed lattice structures. The statistical results showed that the yield strength of the TTN-2 structures is 36 ± 4 MPa, which is almost two times of the solid-strut OCTET sample of 18.2 ± 1.5 MPa. Moreover, the TTN-2 lattice structure also demonstrated slightly higher yield strength than the TAN-2 (32.4 ± 3 MPa) lattice structure. On the other hand, the TTN-2 and TAN-2 lattice structures showed similar Young’s modulus of 0.92 ± 0.04 GPa and 0.88 ± 0.03 GPa, respectively, which both surpassed the solid-strut sample (0.55 ± 0.02 GPa) with the same relative density. Consequently, the gradient hollow-strut lattice structure delivered much higher energy absorption values than that of the solid-strut sample, as the yield stress and the plateau stress of the TTN-2 and TAN-2 lattice structures are much more constant and higher.

**Figure 8:**
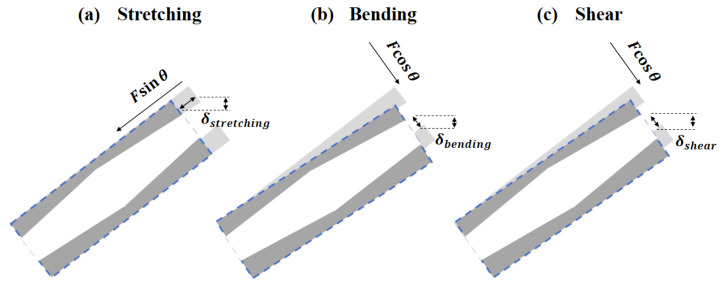
Schematic drawing of the deformation of gradient hollow strut under (**a**) stretching, (**b**) bending and (**c**) shear.

To understand the underlying reasons for the strength enhancement of the gradient hollow-strut lattice structures, a TTN-type strut with a unit length of *L* is analyzed to study its deformation behavior under an externally applied compressive force of *F*, as illustrated in Figure 8. Generally, the deformation is influenced by multiple mechanisms, including stretching, bending and shear modes [33]. The respective displacement of the strut caused by stretching and shear can be expressed as Equations (2) and (3):

(2) $$\delta_{streching}=\frac{LF\sin^{2}\theta }{AE_{s}}.$$

(3) $$\delta_{Shear}=\frac{LF(2+2v)\cos^{2}\theta }{AkE_{s}}.$$

where *A* is the cross-sectional area, *k* is the shear coefficient, *v* is the Poisson’s ratio, and *E_s_* is the Young’s modulus of the material. The cross-sectional area of the solid strut remains constant, while it varies slightly with the change of the geometrical parameters of the gradient hollow strut. Since the strut length and relative density of the lattice structures are fixed, the cross-sectional areas show minimal variance between the solid-strut and the gradient hollow-strut lattice structures; thus, the displacement difference caused by stretching deformation can be considered negligible. On the other hand, the shear coefficient is calculated as 0.8886 for the solid strut, while it ranges from 0.561 to 0.580 with the changes in the cross-sectional area of the gradient hollow strut. However, such a modest decrease in shear coefficient from solid strut to gradient hollow strut only results in a small increase in shear deformation, which has a negligible impact on shear strength [34].

The displacement of the strut induced by bending deformation can be written as:

(4) $$\delta_{Bending}=\frac{L^{3}F\cos^{2}\theta }{12E_{s}I}$$

wherein the second moment inertia *I* for solid (*I_s_*) and hollow (*I_h_*) struts can be calculated by:

(5) $$I_{s}=\frac{\pi d_{solid}^{4}}{64}$$

(6) $$I_{h}=\frac{\pi (d_{outer}^{4}-d_{inner}^{4})}{64}$$

Take the TTN-2 as an example; the *I_s_* is determined as 0.425 mm^4^, while the calculated *I_h_* ranged from 1.727 to 1.921 mm^4^ with the changes of the cross-sectional areas. The substantial increment in the moment of inertia from solid strut to gradient hollow strut will result in a significant decrease in bending deformation, leading to a remarkable increase in the bending strength of the gradient hollow-strut lattice structures. On such basis, we can conclude that the gradient hollow-strut lattice structures are considerably stronger (higher yield strength) and stiffer (higher Young’s modulus) than the solid strut counterpart, primarily due to the significantly increased bending strength. Additionally, the yield strength difference between the TTN-2 and TAN-2 structures is due to the reversal of the maximum and minimum cross-sectional area positions as increments can bear more load. Given the stress concentration at the node areas, the larger thickness of the TTN-2 structure at the node region provides higher load-bearing capacity as compared with the TAN-2 structure, leading to the TTN-2 structure with slightly higher yield strength.

### 3.4. Deformation Mode Analysis

**Figure 9:**
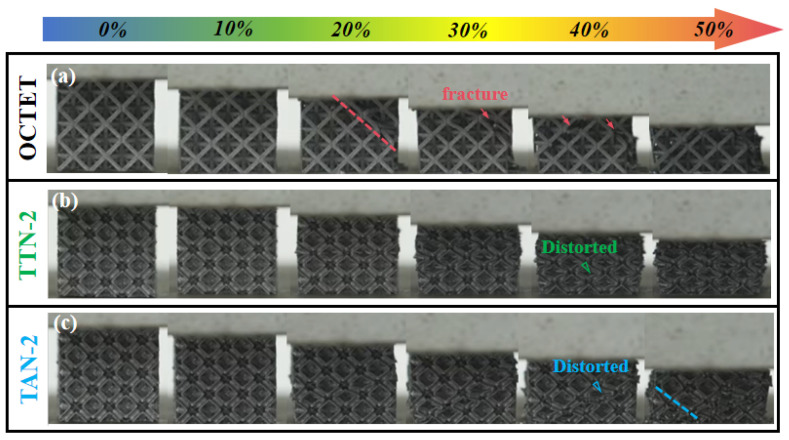
The recorded compression deformation process from 0 to 0.5 strain of the LPBFed (**a**) OCTET, (**b**) TTN-2 and (**c**) TAN-2 lattice structure samples.

Figure 9 shows the recorded deformation process from the initial state to the 50% compression strain of the LPBFed lattice structure samples. The solid-strut OCTET sample experienced obvious shearing deformation at a plastic strain of only 20%, and local fracture and collapse were observed afterward (Figure 9a). The solid-strut metal lattice structures deform via global fracture along the 45° shear plane under uniaxial compression, which was frequently reported [35]. This is primarily due to severe stress concentration at the node area, triggering the 45° direction as the most vulnerable plane for part of the lattice structure slipping. On the other hand, the TTN-2 and TAN-2 octet lattice structures with gradient hollow struts demonstrated significantly enhanced deformation stability, and no obvious shear and/or local fracture was observed for at least 40% compression strain. As indicated by the triangle symbols, it is suggested that the distortion and tilting of the hollow struts successfully enhanced the deformation resistance and stability due to the improved cross-sectional material areas (Figure 9b,c). As the deformation strain increased to 50%, the TAN-2 sample exhibited slight shearing behavior at the bottom left corner, while the TTN-2 maintained the best shape integrity.

**Figure 10:**
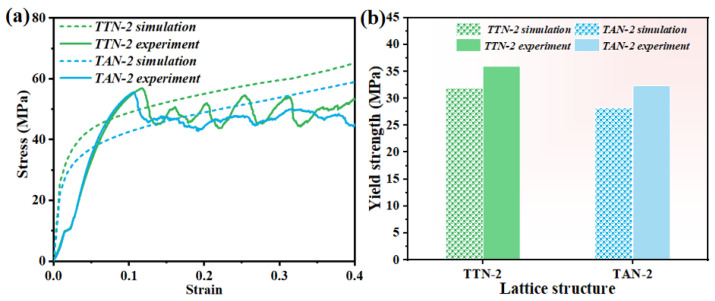
Comparison of the simulation and experimental results of the TTN-2 and TAN-2 lattice structures. (**a**) The stress–strain curves and (**b**) the derived yield strength.

To understand the inherent compression deformation mechanisms, we performed FEA simulations of the studied lattice structure samples. Figure 10 shows the experimentally tested and the FEA-simulated engineering stress–strain curves and mechanical properties of TTN-2 and TAN-2 samples. Due to the uneven surface and hard contact between the lattice structure and the cross-heads of the testing machine, non-linear deformation behavior was observed in the elastic deformation stage. The respective numerical yield strength values of the TTN-2 and TAN-2 samples were determined to be about 32 and 28 MPa, which was close enough to the experimental results of 36 ± 4 MPa and 32.4 ± 3 MPa. The property discrepancy can result from the inconsistency between the manufactured samples and the CAD model, as well as the surface defects observed in Figure 5. Overall, the reasonable agreements between the simulated and measured yield strength validated our numerical model.

**Figure 11:**
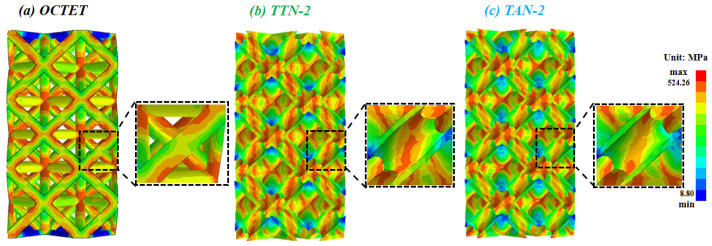
The FEA-predicted stress distribution and the local enlarged view of the studied (**a**) OCTET, (**b**) TTN-2 and (**c**) TAN-2 lattice structures.

The Von Mises stress distribution maps at a compression strain of 5% of the studied lattice structures are presented in Figure 11. It is evident that the stress is mainly concentrated at the node regions for all types of lattice structures. However, as compared with the solid-strut lattice structure, a significant amount of stress was observed on the struts of the TTN-2 and TAN-2 lattice structures, which demonstrated improved stress distribution uniformity (Figure 11b,c). Such observation highlighted the effectiveness of the designed gradient hollow strut in enhancing the loading bearing capacity, which can be attributed to the enhanced bending strength of the gradient hollow struts. Moreover, the designed gradient hollow struts also reduced the risk of buckling due to the increased slenderness ratio as compared to the solid struts. Therefore, the gradient hollow-strut lattice structures exhibited superior performance in terms of structural integrity, whereas the solid lattice was prone to collapse and fracture (Figure 9).

On the other hand, the inner hollow diameter of the TTN-2 strut decreased from the center towards both two ends, and the cross-sectional solid areas of the TTN strut increased towards the two ends. As for the TAN-2 strut structure, it exhibited the opposite trends, i.e., its inner hollow strut diameter increased from the center towards both two ends, and the cross-sectional solid areas decreased towards the two ends. Consequently, as shown in the magnified views of Figure 11b,c, the stress on the middle cross-section of the TTN-2 strut is slightly larger than that of the TAN-2 strut due to the decreased wall thickness at the strut middle area. According to Equations (2)–(4), the increased cross-section area enhanced the stretching, bending and shear strength of the TTN-2 structure at the node regions. These lead to a reasonable improvement in load-bearing capacity and deformation stability of the TTN-2 lattice structure compared to the TAN-2 lattice structure.

## 4. Conclusions

In this study, the bamboo node-inspired hollow-strut octet lattice structures were designed and fabricated by LPBF. The effect of the geometrical parameters of the gradient hollow strut on mechanical properties, deformation modes and energy absorption characteristics was systematically investigated by experimental compression tests and FEA analysis. The main findings of this study can be summarized as follows:

(1) FEA analysis revealed that the higher yield strength, Young’s modulus and energy absorption for the designed octet unit cells were obtained when the shape parameter *k* equals 0.1. The optimized octet unit cells of TTN-2 and TAN-2 were redesigned into lattice structures for experimental studies.
(2) The gradient hollow-strut lattice structure design significantly enhanced its mechanical properties as compared with the solid-strut lattice structures under the same relative density, while the TTN-2 structure exhibited slightly higher yield strength than the TAN-2 structure.
(3) The deformation stability of the TTN-2 and TAN-2 structures was markedly enhanced by suppressing the typical shearing and local failure issues observed in the solid-strut octet lattice structure. Specifically, the TTN-2 structure displayed the best shape integrity even after 50% compression strain.
(4) Deformation modes analysis revealed that the enhanced bending strength due to the gradient hollow strut design enhanced its mechanical performance as compared with the solid strut counterparts. On the other hand, the slight strut cross-section configuration variance contributed to the reasonable improvement in load-bearing capacity and deformation stability of the TTN-2 lattice structure.

The current study confirmed that the mechanical properties and deformation stability of the octet lattice structure can be enhanced by tailoring the geometrical parameters of the hollow strut. The current design strategy can be extended to other strut-based lattice structure designs and optimization for LPBF fabrication. Moreover, significantly enhanced mechanical properties are also expected if the LPBF manufacturing fidelity and/or the surface quality can be further improved, as local stress concentration issues induced by surface roughness can be reduced or even avoided. These will be explored in future studies.

## Figures and Tables

**Table 1:** Geometrical parameters of the developed TTN strut unit cell structures.

Unit Cell	*m* (mm)	*n* (mm)	|*m* − *n*|
TTN-1	0.9	1.0	0.1
TTN-2	0.8	0.9	0.1
TTN-3	0.7	0.9	0.2
TTN-4	0.6	0.9	0.3
TTN-5	0.7	0.8	0.1
TTN-6	0.6	0.8	0.2
TTN-7	0.5	0.8	0.3
TTN-8	0.6	0.7	0.1
TTN-9	0.5	0.7	0.2

**Table 2:** Geometrical parameters of the developed TAN strut unit cell structures.

Unit Cell	*m* (mm)	*n* (mm)	|*m* − *n*|
TAN-1	1.0	0.9	0.1
TAN-2	0.9	0.8	0.1
TAN-3	0.9	0.7	0.2
TAN-4	0.9	0.6	0.3
TAN-5	0.8	0.7	0.1
TAN-6	0.8	0.6	0.2
TAN-7	0.8	0.5	0.3
TAN-8	0.7	0.6	0.1
TAN-9	0.7	0.5	0.2

## Data Availability

The data that support the findings of this study are available from the corresponding authors upon reasonable request.
